# Assessment of the Current Endodontic Practices among General Dental Practitioners in the Kingdom of Saudi Arabia

**DOI:** 10.3390/ijerph19116601

**Published:** 2022-05-28

**Authors:** Rizwan Jouhar, Muhammad Adeel Ahmed, Hussain Abdulmuttalib Ali Almomen, Abdullah Amin Jawad BuHulayqah, Mohammed Yousef Ahmed Alkashi, Ahmed Adel A. Al-Quraini, Naseer Ahmed

**Affiliations:** 1 Department of Restorative Dental Sciences, College of Dentistry, King Faisal University, Al-Ahsa 31982, Saudi Arabia; mshakeel@kfu.edu.sa (M.A.A.); 217033469@student.kfu.edu.sa (H.A.A.A.); 217025619@student.kfu.edu.sa (A.A.J.B.); 217025704@student.kfu.edu.sa (M.Y.A.A.); aalquraini@kfu.edu.sa (A.A.A.A.-Q.); 2 Prosthodontics Unit, School of Dental Sciences, Health Campus, Universiti Sains Malaysia, Kota Bharu 16150, Malaysia; naseerahmed@student.usm.my; 3 Department of Prosthodontics, Altamash Institute of Dental Medicine, Karachi 75500, Pakistan

**Keywords:** endodontic practices, general dental practitioners, questionnaire study, professional experience, undergraduate education

## Abstract

A contemporary knowledge of root canal treatment (RCT) is a prerequisite for a successful outcome. Studies observed that General Dental Practitioners (GDPs) were not abreast of current endodontic knowledge due to a lack of continuing dental education, not following the treatment protocols that they had learned in their undergraduate program, and overlooking the evidence-based current endodontic practices. Therefore, this study was intended to assess the awareness, attitude, and clinical endodontic practices among General Dental Practitioners in Saudi Arabia. This cross-sectional questionnaire-based study was conducted among all 312 GDPs working in Saudi Arabia. The questionnaire consisted of socio-demographic details and 23 questions regarding current endodontic practices. The collected data was analyzed using the SPSS Version 21 (Chicago, IL, USA). The chi-square test was applied to explore the influence of gender, workplace, and the years of professional activity on the materials and techniques employed in the RCT procedure. The study results showed that of all respondents, 159 (51.0%) were males, 153 (49.0%) were females, and 286 (91.7%) were Saudi nationals. Most of the GDPs, i.e., 204 (65.4%) practiced in private hospitals or clinics whereas 108 (34.6%) practiced in Government hospitals. Root canal treatment on all teeth had been performed by 196 (62%) of the practitioners. Association of gender with demographic details and endodontic practices revealed a statistically significant difference between both genders with respect to region, nationality, type of RCT treated on the tooth, and the technique used to measure the working length (*p* < 0.05). Furthermore, years of professional experience and workplace significantly affect endodontic practices (*p* < 0.05). This study concluded that most of the general dental practitioners complied with quality standard guidelines showing a positive attitude toward endodontic practices. Furthermore, irrespective of gender, most of the steps in endodontic procedures revealed a significant association with years of professional experience and the workplace.

## 1. Introduction

The basic goal of endodontic treatment is to eliminate the infection and prevent the root canal system from becoming infected again. For this purpose, strict aseptic procedures and high technical measurements are required [1,2]. It is also evident that the outcomes of root canal treatment are based on various pre-operative, intraoperative, and postoperative clinical factors along with the practitioner’s knowledge, attitude, practices, and education level [3,4].

Effective root-canal treatment relies on cleaning and shaping with appropriate debridement of the root canal system [5,6]. The success of root canal therapy entails a complete mechanical preparation with the help of conventional hand instruments such as reamers, K-files, and Hedstrom files which are frequently used instruments among General Dental Practitioners (GDPs) [7]. Therefore, the treatment’s success depends on accurate chemo-mechanical cleaning to eradicate the pulpal debris, dentinal remnants, and microorganisms consequently removing the etiological causes of endodontic infection. Thus, the root canal instrumentation must always be supplemented by irrigation to eliminate the pulpal remnants. Instrumentation becomes ineffective and remnants are not properly eliminated owing to insufficient irrigation [8,9].

In contemporary endodontics, rubber dam isolation is recognized as the standard of care. In an assessment among American general dental practitioners, 59% of respondents indicated they constantly applied rubber dams for isolation [10].

In the case of intra-canal infection, incorrect determination of canal length leads to over-instrumentation that encourages the dislodgment of septic dentine or debris into the tissues surrounding a root and can compromise healing. Hence, the working length is a very significant aspect in evaluating the excellence of endodontic treatment. Ideally, it is believed that the working length seems to be 1–2 mm from the radiographic apex [11,12]. The inter-appointment medicaments have been promoted to deliver an uninterrupted quantity of antimicrobial agents that limits the growth of bacteria and blocks bacterial multiplication [13]. Generally, a range of intra-canal medicines has been recommended comprising calcium hydroxide, Eugenol, iodine potassium iodide, phenolic compounds, formocresol, and numerous antibiotics [14,15].

However, there is contradictory and unsatisfactory evidence that supports the combination of calcium hydroxide with chlorhexidine improving anti-bacterial properties [16]. In contrast, Zehnder et al. demonstrated that amalgamation of calcium hydroxide with sodium hypochlorite presented considerably enhanced tissue dissolving effects and improved antimicrobial effectiveness than mixed with normal saline [17].

In spite of significant advancements in contemporary endodontics regarding root canal infections, mechanical instrumentation of radicular spaces, and related apical periodontitis lesions stay unusually widespread [18]. Indeed, current systematic analysis has stated a rise in the incidence of apical periodontitis in the last 8–9 years, seemingly owing to unsatisfactory endodontic and restorative management [19]. 

The success of root canal treatment performed by an endodontist in the scientific literature was reported as up to 90% [4]. However, root canal treatment in many places in Saudi Arabia is performed by General Dental Practitioners (GDPs) owing to the fact that qualified endodontists are either not available or unaffordable to many patients while GDPs are easily accessible to patients [20]. Many studies reported that GDPs do not follow the proper treatment guidelines and provide sub-standard treatment; hence, their endodontic treatment success was observed between 65% and 75% [20,21,22]. 

Contemporary knowledge of root canal treatment is a prerequisite for a successful outcome [23]. In the past 15 years, the latest developments in endodontic treatment such as the availability of newer materials, equipment, and techniques have made a significant contribution to raising the predictability of a successful outcome. However, studies observed that GDPs were not abreast of the current endodontic knowledge due to a lack of continuing dental education and not following the treatment protocols that they had learned in their undergraduate program, as well as overlooking the evidence-based current endodontic practices [20,23,24]. Therefore, the aim of this study was to assess the awareness, attitude, and clinical endodontic practices among GDPs in Saudi Arabia.

## 2. Materials and Methods

This cross-sectional questionnaire-based study was conducted among all GDPs working in different government and private hospitals and dental clinics in Saudi Arabia. A well-constructed questionnaire was designed and validated through intra-class correlation with a strong relation of 0.74. The questionnaire was distributed to 374 General Dental Practitioners. Three hundred and twenty-one (321) participants consented to be part of the current study; however, 9 participants were excluded due to incomplete information. Hence, 312 participants were included in this study. The ethical approval of this study was obtained from the committee of scientific research, King Faisal University, Al-Ahsa (KFU-REC-2022-JAN-EA000353). 

The questionnaire consisted of 28 multiple-choice questions. Respondents were asked to choose one suitable answer for the questions. The questionnaire was composed of two sections. The first section comprised socio-demographic information such as age, gender, region (east, west, north, south, and central), citizenship (Saudi\non-Saudi), years of experience (<5 years, 5–10 years, 11–15 years, and >15 years), and workplace (government\private). The second section comprised 23 questions about the practitioner’s endodontic practices. These questions were related to conducting all the necessary investigations for making a diagnosis and asking about the aseptic measures used during the treatment. Further questions were based on the methods used for access cavity, locating the canals, pulp extirpation, use of rubber dams, and isolation methods, and the choice of antibacterial agents and canal irrigants, e.g., shaping and cleaning, obturation, and the coronal seal, etc. 

### Statistical Analysis

The collected data were analyzed using the Statistical Package for the Social Sciences Software (SPSS Statistics, version 25, Chicago, IL, USA). Descriptive statistics were documented as frequencies (*n*) and percentages (%). The chi-square test was applied to explore the influence of gender, workplace, and the years of professional activity on the materials and techniques employed in the RCT procedure. A *p*-value of ≤0.05 was considered significant. 

## 3. Results

A total of 312 respondents participated in this study. Of all respondents, 159 (51.0%) were males and 153 (49.0%) were females. The mean age of the participants was 27.48 ± 2.6 years. Most of the respondents, 286 (91.7%), were Saudi nationals and 26 (8.3%) were non-Saudi. Of all respondents, 133 (42.6%) resided in the eastern region of Saudi Arabia, 61 (19.6%) resided in the western region, 50 (16.0%) resided in the southern region, 19 (6.1%) resided in the northern region, and 49 (15.7%) resided in the central region of Saudi Arabia. Most of the GDPs, i.e., 204 (65.4%) practiced in a private hospital or clinic whereas 108 (34.6%) practiced in a Government hospital. Most of the dental practitioners, i.e., 276 (88.5%) worked in public health care with less than 5 years of experience whereas 26 (8.3%) had working experience of 5–10 years, as shown in Table 1. 

The majority of the practitioners, 196 (62%), performed RCTs in all teeth whereas 88 (28.2%) of practitioners had only performed RCTs in anterior and premolars. Clinically, more than two-thirds, 256 (82.1%), of the respondents were using only cold tests to assess the pulp vitality whereas electric pulp testing was used to assess the pulp vitality by 30 (9.6%) of the respondents. Approximately, more than half of the respondents, 202 (64.7%) were performing RCT in both single and multiple visits. The majority of the respondents, 142 (45.5%), managed flare-ups between the endodontic appointments with the placement of intracanal medicaments. Most of the respondents, 231 (74.0%), preferred the rubber dam isolation method, and 58 (18.6%) applied rubber dams occasionally. Out of all respondents, 220 (70.5%) preferred to use round bur for access cavity preparation, with straight fissure bur preferred by 42 (13.5%) respondents. Additionally, 137 (43.9%) respondents used a visual method and 130 (41.7%) respondents used DG-16 explorer to locate the canals. Removing the pulp tissue by barbed broaches was preferred by 146 (46.8%) respondents followed by K-files by 100 (32.1%) respondents. Radiographic evaluation along with an electronic apex locater was the most commonly used method for working length determination. Most of the respondents used both methods 247 (79.2%). The majority of the respondents, 187 (59.9%), used both rotary and manual instrumentation for cleaning and shaping the canal. Most of the respondents, 189 (60.6%), used patency files to keep apical foramen patent. The most commonly used irrigation solution was sodium hypochlorite, 199 (63.8%), followed by variable irrigants used by 71 (22.8%). As far as the type of irrigation technique is concerned, 165 (52.9%) respondents used a syringe with a side-ended needle followed by a syringe with a regular needle by 134 (42.9%). Most respondents, 268 (85.9%), did not leave the tooth open in infected canals. The majority, 490 (66%) of the respondents preferred a single cone as an obturation technique followed by cold lateral condensation by 94 (30.1%). Cutting the gutta-percha at the orifice level was preferred by 195 (62.5%) of respondents whereas 93 (29.8%) respondents preferred cutting below the orifice. A resin-based root canal sealer was most frequently selected by 158 (50.6%) respondents, a zinc oxide Eugenol sealer by 77 (24.7%), followed by a calcium-hydroxide-based sealer by 56 (17.9%). Most of the dental practitioners 130 (41.7%) preferred to conduct the core buildup immediately after obturation, while some 114 (36.5%) opted to perform it within one week. The most common material used for the core buildup after RCT was composite preferred by 231 (74.0%) respondents followed by GIC used by 47 (15.1%). Of all respondents, 127 (40.7%) performed occlusal reduction after the RCT whereas 121 (38.8%) performed it only occasionally. Concerning extra coronal restoration, 190 (60.9%) of dental practitioners recommended a crown or bridge after root canal treatment. In the case of endodontic mishaps, 183 (58.7%) dental practitioners discontinued the treatment and referred the patient to an endodontist for improvements. Surprisingly, the majority, 142 (45.5%) of the practitioners did not follow up their endodontic cases, as shown in Table 2.

Association of gender with demographic details and endodontic practices among dental practitioners revealed that there was a statistically significant difference between both genders with respect to the region (*p* = 0.024), indicating that most of the males reside in the east of Saudi Arabia. Nationality was also significantly affected by the gender of the dental practitioner (*p* = 0.031). The type of RCT on the tooth was also significantly affected by gender (*p* = 0.010). The preference for barbed broach was slightly significant by gender (*p* = 0.051) with 79 (51.6%) of the respondents using barbed broach being female. There was a statistically significant difference between the genders regarding the technique used to measure the working length of the tooth (*p* = 0.043). On the other hand, pulp vitality, management of flare-ups in between appointments, rubber dam isolation; bur used in cavity preparation, cleaning and shaping of the canal, and obturation technique were not significantly influenced by the gender, as shown in Table 3.

The association of years of professional experience with demographic details and endodontic practices among dental practitioners discovered that the years of professional experience is statistically significantly affected by practitioners’ nationality (*p* < 0.001) indicating most of the practitioners were Saudi nationals with less than 5 years’ experience. The use of intracanal medicaments was slightly influenced by the practitioners’ years of professional experience (*p* = 0.053). It was found that the years of professional experience significantly affect the use of method to locate the canals (*p* < 0.001) showing most of the practitioners with less than 5 years of experience preferred visual only to locate the canals followed by DG-16 explorer. It was observed that years of professional experience significantly influence leaving the tooth open in infected canals (*p* = 0.016) and cutting the gutta-percha at the orifice level (*p* = 0.013). There was a statistically significant difference between the years of professional experience and occlusal reduction after RCT (*p* = 0.033), referring to an endodontist in the case of endodontic mishap (*p* = 0.001), and following up on RCT cases (*p* < 0.001), as shown in Table 4.

As far as the association of the workplace is concerned, demographic details such as region and nationality were significantly affected by the workplace of dental practitioners (*p* = 0.035, *p* = 0.001) respectively. The type of RCT-treated teeth was also significantly influenced by the workplace (*p* = 0.010). Furthermore, there was a statistically significant difference observed between government and private dental practitioners in terms of number of visits to perform RCT (*p* = 0.001), management of flareups (*p* = 0.005), rubber dams for isolation (*p* < 0.001), the cleaning and shaping technique (*p* < 0.001), type of irrigation technique (*p* = 0.019), method of obturation (*p* < 0.001), immediate core buildup after obturation (*p* < 0.001), and follow-up of RCT cases (*p* = 0.034), as shown in Table 5. 

## 4. Discussion

Scientifically, it is evident that there is a number of reasons related to the poor results of root canal treatments, in which intrinsic or extrinsic non-microbial factors, quality of endodontic treatment, extra-radicular and/or intra-radicular contagions, and coronal restoration, are included [25]. For any service, quality is the vital element that does not occur in isolation. Consequently, it is based on the treatment of endodontic standards that are applied by the general dental practitioners in the government and private sectors [26]. 

The current study demonstrated the facts on the preferred choice of the materials, methods, and current trends employed in root canal treatments by Saudi dentists. Out of 312 respondents in this study, almost half of them were males 159 (51.0%) and the remaining half were females 153 (49.0%). The majority, 196 (62%) of the practitioners had performed root canal treatments on all teeth. Further stratification showed that 8 (3.9%) dentists from the private sector and 17 (15.7%) from the government section had performed root canal treatment in anterior teeth only. This difference in the private and government sectors may be due to the fact that the government hospitals are open 24 h for emergency services and perhaps, they received more pediatric patients for root canal treatment in anterior teeth secondary to dental trauma.

Approximately, more than half of the respondents, 202 (64.7%) performed root canal treatment in both single and multiple visits. In addition, rubber dam isolation was used by most of the respondents 231 (74.0%). These findings were inconsistent with the research by Gaikwad A. et al. [27], who surveyed 178 dentists wherein 96 were males and 82 were females and demonstrated that 86.4% performed RCT in posterior teeth only. Their study revealed that cotton rolls were used as the main isolation method (74.6%) and very limited practitioners used rubber dams during an endodontic procedure (3.2%) indicating that the majority of the practitioners did not comply with the required quality standard guiding principles concerning rubber dams.

Endodontic treatment of any tooth is a challenging procedure as its success depends on the accurate cleaning, shaping, and obturation of a canal with appropriate armamentarium along with proper isolation means [28]. The present study revealed that most of the respondents, 247 (79.2%) preferred both a radiograph and an apex locator to determine the working length accurately. Sodium hypochlorite was the best irrigant solution that was used by most of the respondents, 199 (63.8%), a high percentage of participants preferred to debride the canal without activation (95.8%). Concerning a sealer, zinc oxide eugenol sealer 77 (24.7%) followed by calcium-hydroxide-based 56 (17.9%) root canal sealers were most frequently chosen by the respondents. These results were consistent with the survey conducted in Saudi Arabia [29], which proved that most practitioners (63%) used both apex locators and periapical x-ray for measuring working length, (70%) of the practitioners performed irrigation without activation, and (66.7%) preferred zinc oxide eugenol-based sealer.

In the present study, GDPs that implemented the standards of endodontic practice reported work experience of <5 to >15 years that was contrasting to the results of other Saudi research [30], in which it was indicated that the GDPs do not follow quality standards of endodontic guiding principles. Therefore, one more study was conducted to discover their KAP [31]. The study demonstrated that most of the study participants had 6–10 years of experience whereas, in the analysis by Al-Nahlawi et al. [32], it was stated that dental practitioners had >10 years of work experience. Conflicting findings were reported in a study by Bogari et al. [33] in which most study participants were freshly graduated.

Assessment of pulpal status can be a perplexing task for GDPs. Thus, a number of tests are always needed to assure an accurate endodontic diagnosis [34,35]. Dental pulp tests, like cold tests, and the electronic pulp test (EPT), have been frequently applied to assist in endodontic diagnosis [35]. In the present study, it was reported that most of the respondents 256 (82.1%) relied on the cold test alone to check the pulp vitality followed by electric pulp testing which was recommended by only 30 (9.6%) respondents. These findings were not in agreement with the study by Bogari DF et al., who reported that pulp vitality can be accurately assessed by the cold pulp test accompanied by an EPT rather than using one of them alone [33]. They observed that 42.8% of the GDPs use the cold test to endorse their diagnosis of teeth that required RCT, whereas 55.5% believed that percussion is a dependable approach to diagnose RCT, and only 21.4% of GDPs applied perio-probe in order to identify the existence of depth of a pocket around the pretentious tooth, before commencing the process. The results of a positive percussion test can form inflammation at the site of the periapical area [36].

It has always been recommended to use a rubber dam during the management of endodontics for isolation, to increase visibility, prevent risk from instruments’ aspiration or inhalation, and provide protection from contaminated aerosols to GDPs [37]. The present study recommended that rubber dam application is a mandatory step that was preferred by most of the respondents 231 (74.0%), it was supported by the fact that most practitioners were working in the private sector instead of government hospitals. These findings were not corroborated with research conducted in Nepal, [38] in which it was claimed that only 10.97% of GDPs use rubber dams regularly and did not follow the standards of endodontic principles. The results of this study are very much consistent with other studies [37,39].

In endodontics, observing working length has always been the most critical step because it helps in the preparation of bio-mechanical and RCT obturation and supports a better prognosis [40]. The present study reported that working length can be determined accurately by using a radiograph in combination with an Apex locator. On the other hand, no one respondent supported the tactile sensation in order to determine the working length. These findings were not in accordance with the study by Manandhar et al., which demonstrated that most GDPs (96.34%) used radiograph to ascertain working length, however, 6.09% believed in the tactile sensation technique, while 8.53% applied an apex locator followed by radiographic confirmation [38]. This study is consistent with research conducted by Shrestha et al. [41] and Iqbal et al. [24]. According to another study, to find out the working length, the application of tactile sensation was not suggested as the instrument that is being used as it may bind against the wall of the root canal along with their length or may cause perforation apically. To achieve perfect working length; a combination of conventional radiographic methods along with the latest electronic apex locator may be used [42].

Cleaning and shaping of the canal is a sensitive stage that should be done perfectly to get a successful RCT. Of the GDPs, 96.28% used stainless steel hand files, however, only 28.04% and 13.41% of GDPs used hand and rotary nickel-titanium files, respectively [38]. Similarly, the same results have been observed in a study by Shrestha et al. [41], Mehta et al. [43], and Iqbal et al. [24]. Rotary nickel-titanium files allow faster preparation of RCT, reduce canal transportations, and provide greater preservation of tooth structure [44]. Nonetheless, they cannot resolve all clinical conditions and the usage of hand stainless steel files is unavoidable. Our study endorsed the above-mentioned research and indicated that most respondents (63.8%) preferred both manual and instrumentation in order to achieve faster root canal preparation along with greater preservation of tooth structure.

It is important to irrigate the root canals because of accessory canals and the existence of microbes. The perfect irrigant ought to have antimicrobial action as well as the ability of tissue-dissolving properties [45]. The present study revealed that most respondents (63.8%) preferred sodium hypochlorite as it has high tissue liquefying and sanitizing ability followed by normal saline. These results were endorsed by some other studies by Shrestha et al. [41] and Mehta et al. [43], which revealed that the use of sodium hypochlorite and normal saline are the most common irrigants. However, the application of sodium hypochlorite without isolating the area of operation tightly with a rubber dam shows an evidently risky preparation of root canal in the use of potentially irritant irrigation solutions.

A root canal sealer is essential to seal the gap between the obturating core interface and dentinal walls and fill the vacuums and irregularities in the root canal, lateral and accessory canals [37,46]. Lateral compaction of gutta-percha in combination with a root canal sealer is the most extensively recognized method. It is a comparatively simple and multipurpose procedure that has delivered good results and does not require costly equipment [47]. In the present study, obturation of the canal was accomplished by the single cone technique (53.5%) with the integration of a resin-based sealer (50.6%) which is needed to seal the space between the dentinal walls and obturating core interface. These outcomes were not corroborated with the study that showed that the preferred root canal sealer, zinc oxide Eugenol, was applied by 75.6% of GDPs [38].

Consequently, the use of the latest and modern armamentarium has a beneficial impact in order to avoid complications in RCT and support the prevention of intra-radicular and extra-radicular infections.

## 5. Limitations

Despite the strengths of this study which include a good sample size and multiple variables used to assess endodontic practices, the present study has some limitations. The unequal regional distribution can be one of the two possible limitations of this study, the other being a smaller range of age groups selected. Therefore, the outcome of this study should be considered a baseline for further studies within the kingdom with equal regional distribution and also in other countries with a wider age bracket for encompassing experienced dentists. Furthermore, future studies should also focus on insights into contemporary methods applied in clinical endodontics.

## 6. Conclusions

Under the limitation of this study, it is concluded that most of the general dental practitioners complied with quality standard guiding principles showing a positive attitude towards endodontic practices. It has also been observed that the majority of dental practitioners worked in the private sector. Furthermore, irrespective of gender, most of the steps in endodontic procedures revealed a significant association with years of professional experience and the workplace. Moreover, it is suggested for the dentists to further upgrade their awareness and practices with contemporary techniques and use of materials through Continuing Dental Education programs.

## Figures and Tables

**Table 1:** Demographic details of study participants (*n* = 312).

Demographic Variables		*n*	%
Gender	Male	159	51.0
	Female	153	49.0
Region	East of Saudi Arabia	133	42.6
	West of Saudi Arabia	61	19.6
	South of Saudi Arabia	50	16.0
	North of Saudi Arabia	19	6.1
	Central Region	49	15.7
Nationality	Saudi	286	91.7
	Non-Saudi	26	8.3
Years of experience	Less than 5 years	276	88.5
	5–10 years	26	8.3
	10–15 years	3	1.0
	more than 15 years	7	2.2
Workplace	Government	108	34.6
	Private	204	65.4

**Table 2:** Use of various instruments and materials for cleaning, shaping, and obturation in various steps of root canal treatment (RCT).

**What type of teeth do you treat by root canal treatment rct**		
Anterior only	25	8.0
Anterior and premolars	88	28.2
Molars	3	1.0
All teeth	196	62.8
**How do you assess the vitality of pulp to make your diagnosis**		
Hot test	19	6.1
Cold test	256	82.1
Electric pulp testing	30	9.6
Combination of above	7	2.2
**In how many visits do you perform RCT**		
Single visit treatment	26	8.3
Multiple visit treatment	84	26.9
Both	202	64.7
**How do you manage flare-ups in between appointments**		
Occlusal reduction	34	11.2
Antibiotic	57	18.3
Intra canal medicament	142	45.5
Analgesic	61	19.6
Refer to the Specialist	17	5.4
**Do you use rubber dams for isolation**		
Yes	231	74.0
No	23	7.4
Occasionally	58	18.6
**Which bur do you prefer for the access cavity preparation**		
Round	220	70.5
Straight fissure	42	13.5
Tapered bur	31	9.9
Others	19	6.1
**Which method do you use to locate the canal**		
Visual only	137	43.9
DG-16 explorer	130	41.7
Magnification Dyes	32	10.3
CBCT Magnification	8	2.6
Combination of above	5	1.6
**How do you perform pulp extirpation**		
Barbed broach	146	46.8
K-file	100	32.1
H-file	32	10.3
Rotary files	34	10.9
**How do you measure the working length of the tooth**		
Radiograph only	30	9.6
Apex locator only	33	10.6
Both	247	79.2
None	2	0.6
**Which technique do you use for the cleaning and shaping**		
Manual instrumentation	38	12.2
Rotary instrumentation	87	27.9
Both	187	59.9
**Do you keep apical foramen patent by using patency file**		
Yes	189	60.6
No	39	12.5
Occasionally	84	26.9
**What type of irrigation do you use**		
Sodium hypochlorite	199	63.8
EDTA	38	12.2
Chlorhexidine	4	1.3
Combination of above	71	22.8
**What type of irrigation technique do you use**		
Syringe with a regular needle	134	42.9
Syringe with a side ended needle	165	52.9
Activation devices	13	4.2
**Do you leave the tooth open in infected canals**		
Yes	19	6.1
No	268	85.9
Occasionally	25	8.0
**What method of obturation do you use**		
Cold Lateral condensation	94	30.1
Single cone	167	53.5
Warm Vertical condensation	40	12.8
Thermafil	10	3.2
Others	1	0.3
**At what coronal level do you prefer to cut the gutta-percha**		
At the orifice level	195	62.5
Below the orifice	93	29.8
To the pulp chamber level	24	7.7
**What type of sealer do you use**		
Resin-based sealer	158	50.6
Zinc oxide eugenol sealer	77	24.7
Calcium Hydroxide-based sealer	56	17.9
MTA-based sealer	21	6.7
**When do you perform core buildup after obturation**		
Immediately	114	36.5
Within one week	130	41.7
Within two weeks	47	15.1
More than two weeks	21	6.7
**What material do you use for the core buildup after RCT**		
GIC	47	15.1
RMGIC	33	10.6
Composite	231	74.0
Others	1	0.3
**Do you perform occlusal reduction after RCT**		
Yes	127	40.7
No	64	20.5
Occasionally	121	38.8
**Do you advise the patients to get a crown after RCT**		
Yes	190	60.9
No	14	4.5
Occasionally	108	34.6
**What would you do if an endodontic mishap happened**		
Inform the patient	90	28.8
Would not inform the patient	10	3.2
Continue the treatment	13	4.2
Would not inform the patient and continue the treatment	16	5.1
Refer to endodontist	183	58.7
**Do you follow up on your RCT cases**		
No	142	45.5
yes, after every 3 months	91	29.2
yes, after every 6 months	67	21.5
yes, after every 1 year	12	3.8

**Table 3:** Association of demographic profile and endodontic practices with respect to gender.

Variable		Male *n*(%)	Female *n*(%)	*p*-Value
Region	East of Saudi Arabia	80(50.3%)	53(34.6%)	0.024
	West of Saudi Arabia	25(15.7%)	36(23.5%)	
	South of Saudi Arabia	27(17.0%)	23(15.0%)	
	North of Saudi Arabia	6(3.8%)	13(8.5%)	
	Central Region	21(13.2%)	28(18.3%)	
Nationality	Saudi	151(95.0%)	135(88.2%)	0.031
	Non-Saudi	8(5.0%)	18(11.8%)	
Years of experience	Less than 5 years	145(91.2%)	131(85.6%)	0.163
	5–10 years	10(6.3%)	16(10.5%)	
	10–15 years	4(2.5%)	3(2.0%)	
	More than 15 years	0(0.0%)	3(2.0%)	
Work place	Government	57(35.8%)	51(33.3%)	0.641
	Private	102(64.2%)	102(66.7%)	
What type of teeth do you treat by root canal treatment RCT				
	Anterior only	8(5.0%)	17(11.1%)	0.010
	Anterior and premolars	39(24.5%)	49(32.0%)	
	Molars	0(0.0%)	3(2.0%)	
	All teeth	112(70.4%)	84(54.9%)	
How do you assess the vitality of pulp to make your diagnosis				
	Hot test	12(7.5%)	7(4.6%)	0.113
	Cold test	128(80.5%)	128(83.7%)	
	Electric pulp testing	18(11.3%)	12(7.8%)	
	Combination of above	1(0.6%)	6(3.9%)	
In how many visits do you perform RCT				
	Single visit treatment	10(6.3%)	16(10.5%)	0.192
	Multiple visit treatment	39(24.5%)	45(29.4%)	
	Both	110(69.2%)	92(60.1%)	
How do you manage flare-ups in between appointments				
	Occlusal reduction	22(13.8%)	13(8.5%)	0.156
	Antibiotic	34(21.4%)	23(15.0%)	
	Intra canal medicament	64(40.3%)	78(51.0%)	
	Analgesic	32(20.1%)	29(19.0%)	
	Refer to the Specialist	7(4.4%)	10(6.5%)	
Do you use rubber dams for isolation				
	Yes	121(76.1%)	110(71.9%)	0.271
	No	8(5.0%)	15(9.8%)	
	Occasionally	30(18.9%)	28(18.3%)	
Which bur do you prefer for the access cavity preparation				
	Round	119(74.8%)	101(66.0%)	0.148
	Straight fissure	16(10.1%)	26(17.0%)	
	Tapered bur	17(10.7%)	14(9.2%)	
	Others	7(4.4%)	12(7.8%)	
Which method do you use to locate the canal				
	Visual only	70(44.0%)	67(43.8%)	0.079
	DG-16 explorer	68(42.8%)	62(40.5%)	
	Magnification	19(11.9%)	13(8.5%)	
	CBCT	2(1.3%)	6(3.9%)	
	Combination of above	0(0.0%)	5(3.3%)	
How do you perform pulp extirpation				
	Barbed broach	67(42.1%)	79(51.6%)	0.051
	K-file	54(34.0%)	46(30.1%)	
	H-file	14(8.8%)	18(11.8%)	
	Rotary files	24(15.1%)	10(6.5%)	
How do you measure the working length of the tooth				
	Radiograph only	9(5.7%)	21(13.7%)	0.043
	Apex locator only	18(11.3%)	15(9.8%)	
	Both	132(83.0%)	115(75.2%)	
	None	0(0.0%)	2(1.3%)	
Which technique do you use for the cleaning and shaping				
	Manual instrumentation	18(11.3%)	20(13.1%)	0.242
	Rotary instrumentation	51(32.1%)	36(23.5%)	
	Both	90(56.6%)	97(63.4%)	
Do you keep apical foramen patent by using patency file				
	Yes	99(62.3%)	90(58.8%)	0.424
	No	22(13.8%)	17(11.1%)	
	Occasionally	38(23.9%)	46(30.1%)	
What type of irrigation do you use Multiple				
	Sodium hypochlorite	105(66.0%)	94(61.4%)	0.457
	EDTA	21(13.2%)	17(11.1%)	
	Chlorhexidine	1(0.6%)	3(2.0%)	
	Combination of above	32(20.1%)	39(25.5%)	
What type of irrigation technique do you use				
	Syringe with a regular needle	71(44.7%)	63(41.2%)	0.781
	Syringe with a side ended needle	81(50.9%)	84(54.9%)	
	Activation devices	7(4.4%)	6(3.9%)	
Do you leave the tooth open in infected canals				
	Yes	8(5.0%)	11(7.2%)	0.194
	No	142(89.3%)	126(82.4%)	
	Occasionally	9(5.7%)	16(10.5%)	
What method of obturation do you use				
	Cold Lateral condensation	45(28.3%)	49(32.0%)	0.450
	Single cone	92(57.9%)	75(49.0%)	
	Warm Vertical condensation	17(10.7%)	23(15.0%)	
	Thermafil	5(3.1%)	5(3.3%)	
	Others	0(0.0%)	1(0.7%)	
At what coronal level do you prefer to cut the gutta-percha				
	At the orifice level	97(61.0%)	98(64.1%)	0.395
	Below the orifice	52(32.7%)	41(26.8%)	
	To the pulp chamber level	10(6.3%)	14(9.2%)	
What type of sealer do you use				
	Resin-based sealer	85(53.5%)	73(47.7%)	0.183
	Zinc oxide eugenol sealer	43(27.0%)	34(22.2%)	
	Calcium Hydroxide-based sealer	22(13.8%)	34(22.2%)	
	MTA-based sealer	9(5.7%)	12(7.8%)	
When do you perform core buildup after obturation				
	Immediately	51(32.1%)	63(41.2%)	0.111
	Within one week	66(41.5%)	64(41.8%)	
	Within two weeks	31(19.5%)	16(10.5%)	
	More than two weeks	11(6.9%)	10(6.5%)	
What material do you use for the core buildup after RCT				
	GIC	20(12.6%)	27(17.6%)	0.408
	RMGIC	19(11.9%)	14(9.2%)	
	Composite	119(74.8%)	112(73.2%)	
	Others	1(0.6%)	0(0.0%)	
Do you perform occlusal reduction after RCT				
	Yes	59(37.1%)	68(44.4%)	0.381
	No	36(22.6%)	28(18.3%)	
	Occasionally	64(40.3%)	57(37.3%)	
Do you advise the patients to get a crown after RCT				
	Yes	92(57.9%)	98(64.1%)	0.225
	No	10(6.3%)	4(2.6%)	
	Occasionally	57(35.8%)	51(33.3%)	
What would you do if an endodontic mishap happened				
	Inform the patient	51(32.1%)	39(25.5%)	0.672
	Would not inform the patient	6(3.8%)	4(2.6%)	
	Continue the treatment	6(3.8%)	7(4.6%)	
	Would not inform the patient and continue the treatment	7(4.4%)	9(5.9%)	
	Refer to endodontist	89(56.0%)	94(61.4%)	
Do you follow up on your RCT cases				
	No	63(39.6%)	79(51.6%)	0.066
	yes, after every 3 months	47(29.6%)	44(28.8%)	
	yes, after every 6 months	43(27.0%)	24(15.7%)	
	yes, after every 1 year	6(3.8%)	6(3.9%)	

**Table 4:** Association of demographic profile and endodontic practices with respect to years of professional experience.

Variable		Less than 5 Years *n*(%)	5–10 Years *n*(%)	10–15 Years *n*(%)	More than 15 Years *n*(%)	*p*-Value
Gender	Male	146(52.9%)	9(34.6%)	0(0.0%)	4(57.1%)	0.163
	Female	130(47.1%)	17(65.3%)	3(100.0%)	3(42.8%)	
Region	East of Saudi Arabia	117(42.4%)	9(34.6%)	3(100.0%)	4(57.1%)	0.748
	West of Saudi Arabia	54(19.6%)	6(23.1%)	0(0.0%)	1(14.3%)	
	South of Saudi Arabia	44(15.9%)	4(15.4%)	0(0.0%)	2(28.6%)	
	North of Saudi Arabia	18(6.5%)	1(3.8%)	0(0.0%)	0(0.0%)	
	Central Region	43(15.6%)	6(23.1%)	0(0.0%)	0(0.0%)	
Nationality	Saudi	261(94.6%)	19(73.1%)	3(100.0%)	6(85.7%)	<0.001
	Non-Saudi	15(5.4%)	7(26.9%)	0(0.0%)	1(14.3%)	
Workplace	Government	94(34.1%)	11(42.3%)	0(0.0%)	3(42.9%)	0.472
	Private	182(65.9%)	15(57.7%)	3(100%)	4(57.1%)	
What type of teeth do you treat by root canal treatment RCT						
	Anterior only	23(8.3%)	1(3.8%)	0(0.0%)	1(14.3%)	0.062
	Anterior and premolars	75(27.2%)	10(38.5%)	1(33.3%)	2(28.6%)	
	Molars	2(0.7%)	0(0.0%)	0(0.0%)	1(14.3%)	
	All teeth	176(63.8%)	15(57.7%)	2(66.7%)	3(42.9%)	
How do you assess the vitality of pulp to make your diagnosis						
	Hot test	16(5.8%)	3(11.5%)	0(0.0%)	0(0.0%)	0.496
	Cold test	230(83.3%)	18(69.2%)	2(66.7%)	6(85.7%)	
	Electric pulp testing	23(8.3%)	5(19.2%)	1(33.3%)	1(14.3%)	
	Combination of above	7(2.5%)	0(0.0%)	0(0.0%)	0(0.0%)	
In how many visits do you perform RCT						
	Single visit treatment	20(7.2%)	5(19.2%)	0(0.0%)	1(14.3%)	0.261
	Multiple visit treatment	75(27.2%)	6(23.1%)	2(66.7%)	1(14.3%)	
	Both	181(65.6%)	15(57.7%)	1(33.3%)	5(71.4%)	
How do you manage flare-ups in between appointments						
	Occlusal reduction	33(12.0%)	1(3.8%)	0(0.0%)	1(14.3%)	0.053
	Antibiotic	48(17.4%)	8(30.8%)	1(33.3%)	0(0.0%)	
	Intra canal medicament	132(47.8%)	7(26.9%)	0(0.0%)	3(42.9%)	
	Analgesic	50(18.1%)	9(34.6%)	1(33.3%)	1(14.3%)	
	Refer to the Specialist	13(4.7%)	1(3.8%)	1(33.3%)	2(28.6%)	
Do you use rubber dams for isolation						
	Yes	209(75.7%)	16(61.5%)	1(33.3%)	5(71.4%)	0.229
	No	20(7.2%)	3(11.5%)	0(0.0%)	0(0.0%)	
	Occasionally	47(17.0%)	7(26.9%)	2(66.7%)	2(28.6%)	
Which bur do you prefer for the access cavity preparation						
	Round	196(71.0%)	18(69.2%)	0(0.0%)	6(85.7%)	0.344
	Straight fissure	36(13.0%)	4(15.4%)	1(33.3%)	1(14.3%)	
	Tapered bur	28(10.1%)	2(7.7%)	1(33.3%)	0(0.0%)	
	Others	16(5.8%)	2(7.7%)	1(33.3%)	0(0.0%)	
Which method do you use to locate the canal						
	Visual only	122(44.2%)	11(42.3%)	1(33.3%)	3(42.9%)	<0.001
	DG-16 explorer	117(42.4%)	11(42.3%)	0(0.0%)	2(28.6%)	
	Magnification	29(10.5%)	1(3.8%)	1(33.3%)	1(14.3%)	
	CBCT	5(1.8%)	3(11.5%)	0(0.0%)	0(0.0%)	
	Conbination of above	3(1.1%)	0(0.0%)	1(33.3%)	1(14.3%)	
How do you perform pulp extirpation						
	Pulp broach	133(48.2%)	10(38.5%)	0(0.0%)	3(42.9%)	0.533
	K-file	89(32.2%)	7(26.9%)	2(66.7%)	2(28.6%)	
	H-file	26(9.4%)	4(15.4%)	1(33.3%)	1(14.3%)	
	Rotary files	28(10.1%)	5(19.2%)	0(0.0%)	1(14.3%)	
How do you measure the working length of the tooth						
	Radiograph only	26(9.4%)	4(15.4%)	0(0.0%)	0(0.0%)	0.744
	Apex locator only	30(10.9%)	1(3.8%)	0(0.0%)	2(28.6%)	
	Both	218(79.0%)	21(80.8%)	3(100.0%)	5(71.4%)	
	None	2(.7%)	0(0.0%)	0(0.0%)	0(0.0%)	
Which technique do you use for the cleaning and shaping						
	Manual instrumentation	34(12.3%)	2(7.7%)	0(0.0%)	2(28.6%)	0.397
	Rotary instrumentation	81(29.3%)	5(19.2%)	0(0.0%)	1(14.3%)	
	Both	161(58.3%)	19(73.1%)	3(100.0%)	4(57.1%)	
Do you keep apical foramen patent by using patency file						
	Yes	171(62.0%)	14(53.8%)	0(0.0%)	4(57.1%)	0.177
	No	34(12.3%)	4(15.4%)	0(0.0%)	1(14.3%)	
	Occasionally	71(25.7%)	8(30.8%)	3(100.0%)	2(28.6%)	
What type of irrigation do you use						
	Sodium hypochlorite	174(63.0%)	20(76.9%)	2(66.7%)	3(42.9%)	0.753
	EDTA	34(12.3%)	2(7.7%)	1(33.3%)	1(14.3%)	
	Chlorhexidine	4(1.4%)	0(0.0%)	0(0.0%)	0(0.0%)	
	Combination of above	64(23.2%)	4(15.4%)	0(0.0%)	3(42.9%)	
What type of irrigation technique do you use						
	Syringe with regular needle	119(43.1%)	11(42.3%)	1(33.3%)	3(42.9%)	0.962
	Syringe with side ended needle	146(52.9%)	13(50.0%)	2(66.7%)	4(57.1%)	
	Activation devices	11(4.0%)	2(7.7%)	0(0.0%)	0(0.0%)	
Do you leave the tooth open in infected canals						
	Yes	16(5.8%)	1(3.8%)	0(0.0%)	2(28.6%)	0.016
	No	241(87.3%)	19(73.1%)	3(100.0%)	5(71.4%)	
	Occasionally	19(6.9%)	6(23.1%)	0(0.0%)	0(0.0%)	
What method of obturation do you use						
	Cold Lateral condensation	81(29.3%)	7(26.9%)	2(66.7%)	4(57.1%)	0.519
	Single cone	152(55.1%)	14(53.8%)	0(0.0%)	1(14.3%)	
	Warm Vertical condensation	34(12.3%)	4(15.4%)	1(33.3%)	1(14.3%)	
	Thermafil	8(2.9%)	1(3.8%)	0(0.0%)	1(14.3%)	
	Others	1(0.4%)	0(0.0%)	0(0.0%)	0(0.0%)	
At what coronal level do you prefer to cut the gutta-percha						
	At the orifice level	179(64.9%)	11(42.3%)	2(66.7%)	3(42.9%)	0.013
	Below the orifice	75(27.2%)	15(57.7%)	1(33.3%)	2(28.6%)	
	To the pulp chamber level	22(8.0%)	0(0.0%)	0(0.0%)	2(28.6%)	
What type of sealer do you use						
	Resin-based sealer	141(51.1%)	14(53.8%)	1(33.3%)	2(28.6%)	0.551
	Zinc oxide eugenol sealer	68(24.6%)	6(23.1%)	0(0.0%)	3(42.9%)	
	Calcium Hydroxide- based sealer	48(17.4%)	5(19.2%)	2(66.7%)	1(14.3%)	
	MTA-based sealer	19(6.9%)	1(3.8%)	0(0.0%)	1(14.3%)	
When do you perform core buildup after obturation						
	Immediately	106(38.4%)	5(19.2%)	1(33.3%)	2(28.6%)	0.247
	Within one week	114(41.3%)	12(46.2%)	2(66.7%)	2(28.6%)	
	Within two weeks	40(14.5%)	6(23.1%)	0(0.0%)	1(14.3%)	
	More than two weeks	16(5.8%)	3(11.5%)	0(0.0%)	2(28.6%)	
What material do you use for the core buildup after RCT						
	GIC	39(14.1%)	6(23.1%)	0(0.0%)	2(28.6%)	0.886
	RMGIC	30(10.9%)	3(11.5%)	0(0.0%)	0(0.0%)	
	Composite	206(74.6%)	17(65.4%)	3(100.0%)	5(71.4%)	
	Others	1(0.4%)	0(0.0%)	0(0.0%)	0(0.0%)	
Do you perform occlusal reduction after RCT						
	Yes	116(42.0%)	9(34.6%)	0(0.0%)	2(28.6%)	0.033
	No	53(19.2%)	7(26.9%)	3(100.0%)	1(14.3%)	
	Occasionally	107(38.8%)	10(38.5%)	0(0.0%)	4(57.1%)	
	No	31(11.2%)	5(19.2%)	0(0.0%)	2(28.6%)	
	Occasionally, Depending on the case	179(64.9%)	14(53.8%)	2(66.7%)	3(42.9%)	
Do you advise the patients to get a crown after RCT						
	Yes	169(61.2%)	15(57.7%)	2(66.7%)	4(57.1%)	0.205
	No	10(3.6%)	4(15.4%)	0(0.0%)	0(0.0%)	
	Occasionally	97(35.1%)	7(26.9%)	1(33.3%)	3(42.9%)	
What would you do if an endodontic mishap happened						
	Inform the patient	79(28.6%)	10(38.5%)	0(0.0%)	1(14.3%)	0.001
	Would not inform the patient	10(3.6%)	1(3.8%)	0(0.0%)	0(0.0%)	
	Continue the treatment	9(3.3%)	0(0.0%)	0(0.0%)	3(42.9%)	
	Would not inform the patient and continue the treatment	13(4.7%)	3(11.5%)	0(0.0%)	0(0.0%)	
	Refer to endodontist	165(59.8%)	12(46.2%)	3(100.0%)	3(42.9%)	
Do you follow up on your RCT cases						
	No	131(47.5%)	10(38.5%)	1(33.3%)	1(14.3%)	<0.001
	yes, after every 3 months	78(28.3%)	10(38.5%)	0(0.0%)	2(28.6%)	
	yes, after every 6 months	58(21.0%)	5(19.2%)	2(66.7%)	4(57.1%)	
	yes, after every 1 year	9(3.3%)	1(3.8%)	0(0.0%)	0(0.0%)	

**Table 5:** Association of demographic profile and endodontic practices with respect to the workplace.

Variable		Government *n*(%)	Private *n*(%)	*p*-Value
Gender	Male	57(52.8%)	102(50.0%)	0.641
	Female	51(47.2%)	102(50.0%)	
Region	East of Saudi Arabia	52(48.1%)	81(39.7%)	0.035
	West of Saudi Arabia	16(14.8%)	45(22.1%)	
	South of Saudi Arabia	23(21.3%)	27(13.2%)	
	North of Saudi Arabia	7(6.5%)	12(5.9%)	
	Central Region	10(9.3%)	39(19.1%)	
Nationality	Saudi	107(99.1%)	179(87.7%)	0.001
	Non-Saudi	1(0.9%)	25(12.3%)	
Years of experience	Less than 5 years	94(87.0%)	182(89.2%)	0.472
	5–10 years	11(10.2%)	15(7.4%)	
	10–15 years	0(0.0%)	3(1.5%)	
	More than 15 years	3(2.8%)	4(2.0%)	
What type of teeth do you treat by root canal treatment RCT				
	Anterior only	17(15.7%)	8(3.9%)	0.001
	Anterior and premolars	35(32.4%)	53(26.0%)	
	Molars	1(0.9%)	2(1.0%)	
	All teeth	55(50.9%)	141(69.1%)	
How do you assess the vitality of pulp to make your diagnosis				
	Hot test	5(4.6%)	14(6.9%)	0.135
	Cold test	85(78.7%)	171(83.8%)	
	Electric pulp testing	16(14.8%)	14(6.9%)	
	Conbination of above	2(1.9%)	5(2.5%)	
In how many visits do you perform RCT				
	Single visit treatment	9(8.3%)	17(8.3%)	0.001
	Multiple visit treatment	43(39.8%)	41(20.1%)	
	Both	56(51.9%)	146(71.6%)	
How do you manage flareups in between appointments				
	Occlusal reduction	6(5.6%)	29(14.2%)	0.005
	Antibiotic	24(22.2%)	33(16.2%)	
	Intra canal medicament	57(52.8%)	85(41.7%)	
	Analgesic	12(11.1%)	49(24.0%)	
	Refer to the Specialist	9(8.3%)	8(3.9%)	
Do you use rubber dams for isolation				
	Yes	67(62.0%)	164(80.4%)	<0.001
	No	16(14.8%)	7(3.4%)	
	Occasionally	25(23.1%)	33(16.2%)	
Which bur do you prefer for the access cavity preparation				
	Round	75(69.4%)	145(71.1%)	0.327
	Straight fissure	13(12.0%)	29(14.2%)	
	Tapered bur	15(13.9%)	16(7.8%)	
	Others	5(4.6%)	14(6.9%)	
Which method do you use to locate the canal				
	Visual only	52(48.1%)	85(41.7%)	0.309
	DG-16 explorer	46(42.6%	84(41.2%)	
	Magnification	7(6.5%)	25(12.3%)	
	CBCT	1(0.9%)	7(3.4%)	
	Combination of above	2(1.9%)	3(1.5%)	
How do you perform pulp extirpation				
	Barbed broach	54(50.0%)	92(45.1%)	0.420
	K-file	36(33.3%)	64(31.4%)	
	H-file	7(6.5%)	25(12.3%)	
	Rotary files	11(10.2%)	23(11.3%)	
How do you measure the working length of the tooth				
	Radiograph only	14(13.0%)	16(7.8%)	0.207
	Apex locator only	8(7.4%)	25(12.3%)	
	Both	86(79.6%)	161(78.9%)	
	None	0(0.0%)	2(1.0%)	
Which technique do you use for the cleaning and shaping				
	Manual instrumentation	29(26.9%)	9(4.4%)	<0.001
	Rotary instrumentation	16(14.8%)	71(34.8%)	
	Both	63(58.3%)	124(60.8%)	
Do you keep apical foramen patent by using patency file				
	Yes	65(60.2%)	124(60.8%)	0.091
	No	19(17.6%)	20(9.8%)	
	Occasionally	24(22.2%)	60(29.4%)	
What type of irrigation do you use				
	Sodium hypochlorite	66(61.1%)	133(65.2%)	0.142
	EDTA	10(9.3%)	28(13.7%)	
	Chlorhexidine	3(2.8%)	1(0.5%)	
	Combination of the above	29(26.9%)	42(20.6%)	
What type of irrigation technique do you use				
	Syringe with a regular needle	58(53.7%)	76(37.3%)	0.019
	Syringe with a side ended needle	47(43.5%)	118(57.8%)	
	Activation devices	3(2.8%)	10(4.9%)	
Do you leave the tooth open in infected canals				
	Yes	5(4.6%)	14(6.9%)	0.458
	No	92(85.2%)	176(86.3%)	
	Occasionally	11(10.2%)	14(6.9%)	
What method of obturation do you use				
	Cold Lateral condensation	43(39.8%)	51(25.0%)	<0.001
	Single cone	38(35.2%)	129(63.2%)	
	Warm Vertical condensation	23(21.3%)	17(8.3%)	
	Thermafill	3(2.8%)	7(3.4%)	
	Others	1(0.9%)	0(0.0%)	
At what coronal level do you prefer to cut the gutta-percha				
	At the orifice level	64(59.3%)	131(64.2%)	0.611
	Below the orifice	36(33.3%)	57(27.9%)	
	To the pulp chamber level	8(7.4%)	16(7.8%)	
What type of sealer do you use				
	Resin-based sealer	54(50.0%)	104(51.0%)	0.067
	Zinc oxide eugenol sealer	31(28.7%)	46(22.5%)	
	Calcium Hydroxide-based sealer	21(19.4%)	35(17.2%)	
	MTA-based sealer	2(1.9%)	19(9.3%)	
When do you perform core buildup after obturation				
	Immediately	27(25.0%)	87(42.6%)	<0.001
	Within one week	35(32.4%)	95(46.6%)	
	Within two weeks	30(27.8%)	17(8.3%)	
	More than two weeks	16(14.8%)	5(2.5%)	
What material do you use for the core buildup after RCT				
	GIC	19(17.6%)	28(13.7%)	0.678
	RMGIC	10(9.3%)	23(11.3%)	
	Composite	79(73.1%)	152(74.5%)	
	Others	0(0.0%)	1(0.5%)	
Do you perform occlusal reduction after RCT				
	Yes	37(34.3%)	90(44.1%)	0.085
	No	29(26.9%)	35(17.2%)	
	Occasionally	42(38.9%)	79(38.7%)	
Do you advise the patients to get a crown after RCT				
	Yes	61(56.5%)	129(63.2%)	0.050
	No	9(8.3%)	5(2.5%)	
	Occasionally	38(35.2%)	70(34.3%)	
What would you do if an endodontic mishap happened				
	Inform the patient	37(34.3%)	53(26.0%)	0.558
	Would not inform the patient	4(3.7%)	6(2.9%)	
	Continue the treatment	4(3.7%)	9(4.4%)	
	Would not inform the patient and continue the treatment	4(3.7%)	12(5.9%)	
	Refer to endodontics	59(54.6%)	124(60.8%)	
Do you follow up on your RCT cases				
	No	61(56.5%)	81(39.7%)	0.034
	yes, after every 3 months	23(21.3%)	68(33.3%)	
	yes, after every 6 months	21(19.4%)	46(22.5%)	
	yes, after every 1 year	3(2.8%)	9(4.4%)	

## Data Availability

Not applicable.
